# Maternal blood count parameters of chronic inflammation by gestational age and their associations with risk of preterm delivery in the Japan Environment and Children’s Study

**DOI:** 10.1038/s41598-021-93101-2

**Published:** 2021-07-30

**Authors:** Naho Morisaki, Aurélie Piedvache, Chie Nagata, Takehiro Michikawa, Seiichi Morokuma, Kiyoko Kato, Masafumi Sanefuji, Eiji Shibata, Mayumi Tsuji, Masayuki Shimono, Shouichi Ohga, Koichi Kusuhara, Japan Environment and Children’s Study Group, Michihiro Kamijima, Shin Yamazaki, Yukihiro Ohya, Reiko Kishi, Nobuo Yaegashi, Koichi Hashimoto, Chisato Mori, Shuichi Ito, Zentaro Yamagata, Hidekuni Inadera, Takeo Nakayama, Hiroyasu Iso, Masayuki Shima, Youichi Kurozawa, Narufumi Suganuma, Takahiko Katoh

**Affiliations:** 1 Department of Social Medicine, National Center for Child Health and Development, Tokyo, Japan; 2 Department of Education for Clinical Research, National Center for Child Health and Development, Tokyo, Japan; 3 Department of Environmental and Occupational Health, School of Medicine, Toho University, Tokyo, Japan; 4 Department of Health Sciences, Graduate School of Medical Sciences, Kyushu University, Fukuoka, Japan; 5 Department of Obstetrics and Gynecology, Graduate School of Medical Sciences, Kyushu University, Fukuoka, Japan; 6 Research Center for Environmental and Developmental Medical Sciences, Kyushu University, Fukuoka, Japan; 7 Regional Center for Japan Environment and Children’s Study (JECS), University of Occupational and Environmental Health, Kitakyushu, Fukuoka Japan; 8 Department of Environmental Health, School of Medicine, University of Occupational and Environmental Health, Kitakyushu, Fukuoka Japan; 9 Department of Pediatrics, Graduate School of Medical Sciences, Kyushu University, Fukuoka, Japan; 10 Department of Pediatrics, School of Medicine, University of Occupational and Environmental Health, Kitakyushu, Fukuoka Japan; 11 Graduate School of Medical Sciences, Department of Occupational and Environmental Health, Nagoya City University, Nagoya, Japan; 12 National Institute for Environmental Studies, Tsukuba, Japan; 13 National Center for Child Health and Development, Tokyo, Japan; 14 Hokkaido University, Sapporo, Japan; 15 Tohoku University, Sendai, Japan; 16 Fukushima Medical University, Fukushima, Japan; 17 Chiba University, Chiba, Japan; 18 Yokohama City University, Yokohama, Japan; 19 University of Yamanashi, Chuo, Japan; 20 University of Toyama, Toyama, Japan; 21 Kyoto University, Kyoto, Japan; 22 Osaka University, Suita, Japan; 23 Hyogo College of Medicine, Nishinomiya, Japan; 24 Tottori University, Yonago, Japan; 25 Kochi University, Nankoku, Japan; 26 Kumamoto University, Kumamoto, Japan

**Keywords:** Epidemiology, Outcomes research, Pregnancy outcome

## Abstract

Neutrophil-to-lymphocyte ratio (NLR), platelet-to-lymphocyte ratio (PLR) and lymphocyte-to-monocyte ratio (LMR), are three reportedly predictive biomarkers that reflect subclinical chronic inflammatory burden. However, how these biomarkers change during pregnancy and its clinical utility among pregnant women have been rarely studied. Among 76,853 singleton pregnancies delivered at 28–41 weeks of gestation that were enrolled in the Japan Environment and Children’s Study, we observed the distribution of maternal NLR, PLR, and LMR values from week 0 to week 36 using spline curves, as well as their predictive values for preterm delivery with and without hypertensive disorders in pregnancy, placental abruption and intrauterine growth restriction (collectively termed ischemic placental disease due to their shared pathological and pathophysiological features) for measurements at 8–11 weeks, 12–17 weeks, and 18–21 weeks. NLR and PLR increased, whereas LMR decreased, with increasing gestation. High LMR and low NLR observed at 18–21 weeks, but not at earlier gestations, were associated with higher risk of preterm delivery with IPD (odds ratio 1.80 [95% CI 1.02, 3.19] per log[LMR]; odds ratio 0.49 [95% CI 0.29, 0.82] per log[NLR]). All parameters were not predictive of preterm delivery without IPD. We provide a robust reference curve for maternal blood count parameters NLR, PLR, and LMR by gestational week.

## Introduction

Preterm delivery, defined as delivery before 37 completed weeks of gestation, is the leading cause of neonatal mortality and morbidity in many countries^1–3^. Preterm delivery is a multi-factorial syndrome with a variety of causes and underlying factors^4^, including preterm labor as well as maternal or fetal complications requiring medical intervention to terminate pregnancy. A growing body of evidence indicates that inflammation (both clinical and subclinical) is likely playing an important role in triggering preterm labor or developing pregnancy complications leading to preterm delivery^5,6^; therefore, a variety of maternal inflammation-related biomarkers have been studied as potential predictors of preterm delivery^7–9^.

Neutrophil-to-lymphocyte ratio (NLR), platelet-to-lymphocyte ratio (PLR), and lymphocyte-to-monocyte ratio (LMR) are three biomarkers predictive of systemic inflammation which have recently gained interest, as they are widely available markers which can be calculated from simple blood counts and show prognostic significance for several diseases related to chronic low-grade inflammation, including cardiovascular diseases^10,11^ and malignancies^12–14^. However, in the field of obstetrics, research concerning their natural course over the pregnancy period, as well as their predictive values for pregnancy outcomes during pregnancy has been scarce. To the best of our knowledge, only two relatively small case control studies, with 108 and 783 subjects, have investigated the predictive values of these biomarkers for birth outcomes. Akgun et al.^15^ reported that PLR and NLR values obtained within the one month prior to active labor were both negatively correlated with birthweight and gestational age. Daglar^16^ reported that increased LMR levels observed at admission for threatened preterm labor were associated with future preterm delivery. These studies had limited sample size, failed to take into account the differences in background maternal characteristics, and did not incorporate gestational age at measurement into the analysis. We show in our study that these factors have a large effect on observed values of blood count parameters. Therefore, we aimed to explore the association between preterm delivery and the blood count parameters NLR, PLR, and LMR.

## Results

From week 8 to week 36, medians of NLR and PLR measurements increased with gestation whereas medians of LMR measurements decreased. For NLR, measurements before 13 weeks had a median of 3.64 [IQR 2.92, 4.51], for measurements at 13–17 weeks, 4.01 [IQR 3.26, 4.95], and for measurements at 18–21 weeks, 4.41 [IQR 3.60, 5.39]. Similarly, the medians of PLR measurements at these three time periods were 1.23 [IQR 0.99, 1.52], 1.32 [IQR 1.07, 1.63], and 1.43 [IQR 1.16, 1.76], respectively. The medians of LMR measurements were 4.07 [IQR 3.29, 5.00], 4.00 [IQR 3.25, 4.88], and 3.75 [IQR 3.06, 4.58], respectively. These biomarkers were also significantly associated with maternal age, pre-pregnancy BMI, history of previous pregnancy, maternal education, maternal smoking status, household income, and conception method. PLR and LMR were also associated with marital status, whereas NLR was associated with infant sex (Table 1).

**Table 1:** Blood count parameter values by maternal characteristics.

	NLR	p-value*	PLR	p-value*	LMR	p-value*
	Median [IQR]		Median [IQR]		Median [IQR]	
**Fetus characteristic**						
Sex		0.014		0.643		0.570
Male	4.02 [3.25; 4.98]		167.92 [139.49; 203.58]		3.95 [3.22; 4.83]	
Female	4.05 [3.27; 5.00]		168.17 [139.54; 203.94]		3.96 [3.21; 4.84]	
**Mother’s characteristics**						
Age		< 0.001		< 0.001		< 0.001
< 25	3.97 [3.22; 4.91]		162.14 [134.27; 196.28]		4.00 [3.24; 4.84]	
25–29	3.99 [3.22; 4.92]		164.02 [136.38; 199.10]		4.00 [3.25; 4.91]	
30–34	4.03 [3.26; 4.99]		167.96 [139.58; 203.57]		3.98 [3.24; 4.86]	
> 34	4.13 [3.33; 5.09]		174.64 [144.61; 211.11]		3.86 [3.15; 4.72]	
BMI before pregnancy		< 0.001		< 0.001		< 0.001
Under 18.5	4.17 [3.33; 5.13]		170.45 [141.21; 206.15]		3.88 [3.15; 4.76]	
18.5 to 24.9	4.04 [3.27; 5.01]		168.07 [139.63; 203.99]		3.95 [3.21; 4.83]	
25 and over	3.82 [3.12; 4.68]		164.07 [136.05; 198.51]		4.13 [3.38; 5.02]	
Parity and previous delivery		< 0.001		< 0.001		< 0.001
Primipara	4.21 [3.40; 5.19]		169.02 [140.42; 204.66]		3.74 [3.06; 4.56]	
Multipara with previous preterm birth	3.98 [3.24; 4.92]		168.97 [141.18; 203.89]		4.09 [3.30; 5.04]	
Multipara with only term births	3.90 [3.16; 4.81]		167.27 [138.64; 203.10]		4.14 [3.36; 5.04]	
**Life condition**						
Mother education		< 0.001		< 0.001		< 0.001
Middle/high school	3.97 [3.21; 4.91]		165.54 [137.45; 201.08]		4.00 [3.25; 4.88]	
Training	4.02 [3.25; 4.97]		168.20 [139.72; 203.29]		3.98 [3.23; 4.88]	
College	4.08 [3.29; 5.05]		170.78 [141.87; 206.66]		3.90 [3.17; 4.77]	
University/higher	4.13 [3.33; 5.09]		169.39 [140.85; 205.92]		3.88 [3.17; 4.76]	
Partner education		< 0.001		< 0.001		< 0.001
Middle/high school	3.97 [3.22; 4.93]		166.29 [138.01; 202.03]		4.00 [3.24; 4.89]	
Training	4.05 [3.27; 4.99]		169.61 [140.24; 204.72]		3.95 [3.22; 4.83]	
College	4.06 [3.29; 5.03]		167.62 [138.32; 202.32]		4.03 [3.25; 4.85]	
University/higher	4.11 [3.31; 5.07]		169.36 [141.14; 205.53]		3.89 [3.18; 4.76]	
Income		< 0.001		< 0.001		< 0.001
To 200	3.93 [3.18; 4.88]		163.88 [136.61; 197.80]		4.05 [3.27; 4.97]	
200 to 400	4.00 [3.24; 4.94]		166.95 [138.27; 202.41]		4.00 [3.24; 4.88]	
400 to 600	4.05 [3.28; 5.01]		168.64 [140.06; 204.68]		3.95 [3.21; 4.83]	
600 to 800	4.08 [3.27; 5.04]		169.35 [140.56; 204.88]		3.92 [3.18; 4.78]	
Above 800	4.10 [3.29; 5.04]		169.76 [141.13; 205.69]		3.88 [3.18; 4.75]	
No information	4.08 [3.29; 5.06]		168.24 [140.11; 205.48]		3.89 [3.16; 4.77]	
Mother smoking		< 0.001		< 0.001		< 0.001
Never smoker	4.11 [3.32; 5.07]		170.86 [142.19; 207.03]		3.90 [3.18; 4.77]	
Stopped before pregnancy	3.96 [3.19; 4.89]		168.34 [139.52; 202.97]		4.06 [3.29; 4.96]	
Stopped because of pregnancy	3.93 [3.19; 4.88]		160.65 [133.34; 194.54]		3.97 [3.22; 4.81]	
Continued smoking	3.83 [3.14; 4.73]		154.58 [126.58; 188.27]		4.13 [3.35; 5.07]	
Marital status		0.867		< 0.001		< 0.001
Married	4.04 [3.26; 4.99]		168.11 [139.64; 203.92]		3.96 [3.22; 4.84]	
Unmarried	4.03 [3.28; 5.01]		166.40 [136.29; 200.46]		3.88 [3.14; 4.73]	
Conception method		< 0.001		< 0.001		< 0.001
Natural	4.03 [3.25; 4.98]		167.89 [139.50; 203.57]		3.97 [3.22; 4.85]	
Hormone injections	4.12 [3.32; 5.05]		166.46 [136.35; 204.01]		3.87 [3.16; 4.69]	
IVF	4.17 [3.36; 5.16]		172.08 [141.88; 208.02]		3.79 [3.10; 4.63]	
**Timing of blood test**		< 0.001		< 0.001		< 0.001
8–12 weeks	3.63 [2.92; 4.51]		160.01 [133.36; 194.26]		4.07 [3.30; 5.00]	
13–17 weeks	4.01 [3.26; 4.95]		167.90 [139.47; 203.33]		4.00 [3.26; 4.89]	
18–21 weeks	4.41 [3.60; 5.39]		175.00 [144.87; 211.34]		3.75 [3.06; 4.58]	

*Kruskal–Wallis test.

**Figure 1:**
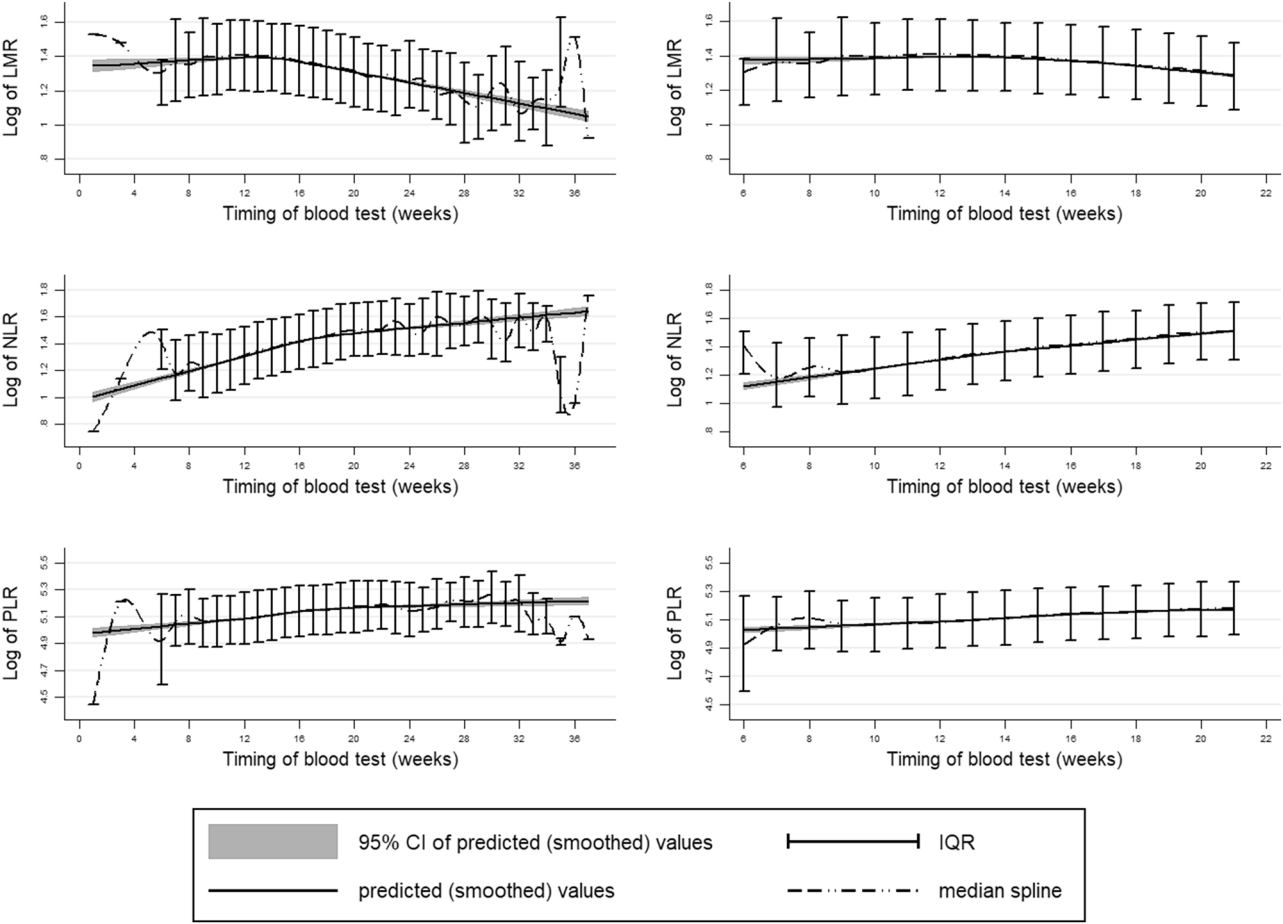
Distribution (median + IQR) and predicted values of each biomarker among all gestations (right) and preterm delivery (left).

Smoothed spline curves on the distribution and predicted values of each biomarker for all weeks of gestation if at least one measurement was available (week 1 to week 36) are shown in Fig. 1. The predicted median of LMR was fairly stable until week 14 when it started to decline and the predicted medians of NLR and PLR increased steadily over all gestations.

Among our participants 3358 delivered preterm (4.5%), including 808 (1.1%) which were preterm delivery with PROM, and 856 (1.2%) were preterm delivery with IPD (5926 (7.9%) with SGA, 1012 (1.4%) with hypertensive disorders in pregnancy and 173 (0.2%) with placental abruption). Associations between the biomarkers (log-transformed values) and preterm delivery as well as sub-types of preterm delivery (preterm delivery with PROM, preterm delivery with IPD) derived from logistic regression models estimating increase in odds for increase in unit (log-values of measurements) are shown in Table 2. NLR and LMR measured at 18–21 weeks showed significant associations with risk of preterm delivery with IPD, with higher NLR associated with lower risk (odds ratio 0.49 [95% CI 0.29, 0.82] per log[NLR]), and higher LMR associated with higher risk (odds ratio 1.80 [95% CI 1.02, 3.19] per log[LMR]). PLR measured at 13–17 weeks was the only biomarker that showed significant association with risk of preterm delivery due to all causes, with higher PLR associated with higher risk (odds ratio 1.19 [95% CI 1.01, 1.41] per log[PLR]). None of the biomarkers measured at 8–12 weeks showed significant associations with risk of overall preterm delivery or preterm delivery with IPD or PROM.

**Table 2:** Biomarker values at early pregnancy and risk of preterm delivery, stratified by timing of blood test.

	8–12 weeks		13–17 weeks		18–21 weeks	
	Crude OR [95% CI]	Adjusted* OR [95% CI]	Crude OR [95% CI]	Adjusted* OR [95% CI]	Crude OR [95% CI]	Adjusted* OR [95% CI]
**Neutrophil to lymphocyte ratio**						
Preterm delivery	0.96 [0.75; 1.23]	0.98 [0.76; 1.26]	1.08 [0.94; 1.25]	1.07 [0.93; 1.25]	0.85 [0.67; 1.08]	0.87 [0.69; 1.11]
Preterm delivery with PROM	1.60 [0.99; 2.59]	1.56 [0.96; 2.53]	1.28 [0.94; 1.73]	1.22 [0.90; 1.66]	1.07 [0.67; 1.69]	1.07 [0.67; 1.71]
Preterm delivery with IPD	1.13 [0.62; 2.06]	1.10 [0.60; 2.01]	1.18 [0.83; 1.68]	1.17 [0.82; 1.67]	**0.52 [0.31; 0.86]**	**0.49 [0.29; 0.82]**
**Platelet to lymphocyte ratio**						
Preterm delivery	0.97 [0.73; 1.29]	0.93 [0.69; 1.24]	**1.23 [1.05; 1.45]**	**1.19 [1.01; 1.41]**	1.02 [0.78; 1.33]	1.01 [0.77; 1.32]
Preterm delivery with PROM	1.28 [0.74; 2.21]	1.26 [0.73; 2.19]	0.95 [0.68; 1.33]	0.91 [0.65; 1.28]	1.48 [0.89; 2.46]	1.47 [0.88; 2.46]
Preterm delivery with IPD	1.22 [0.61; 2.42]	1.05 [0.52; 2.11]	1.32 [0.89; 1.96]	1.22 [0.82; 1.80]	0.92 [0.51; 1.66]	0.85 [0.46; 1.54]
**Lymphocyte to monocyte ratio**						
Preterm delivery	0.96 [0.73; 1.25]	0.95 [0.72; 1.25]	0.99 [0.85; 1.15]	1.01 [0.87; 1.18]	1.15 [0.90; 1.48]	1.12 [0.87; 1.45]
Preterm delivery with PROM	0.80 [0.48; 1.33]	0.87 [0.52; 1.46]	0.81 [0.59; 1.11]	0.88 [0.64; 1.21]	0.87 [0.54; 1.39]	0.88 [0.54; 1.42]
Preterm delivery with IPD	0.75 [0.40; 1.43]	0.79 [0.41; 1.51]	0.81 [0.57; 1.17]	0.85 [0.58; 1.23]	1.72 [0.98; 3.00]	**1.80 [1.02; 3.19]**

*PROM* premature rupture of membranes, *IPD* ischemic placental disease.

*Adjusted model for sex of fetus, mother age, pre-pregnancy BMI, parity, family income, education level of couple, smoking of women and conception method.

**Figure 2:**
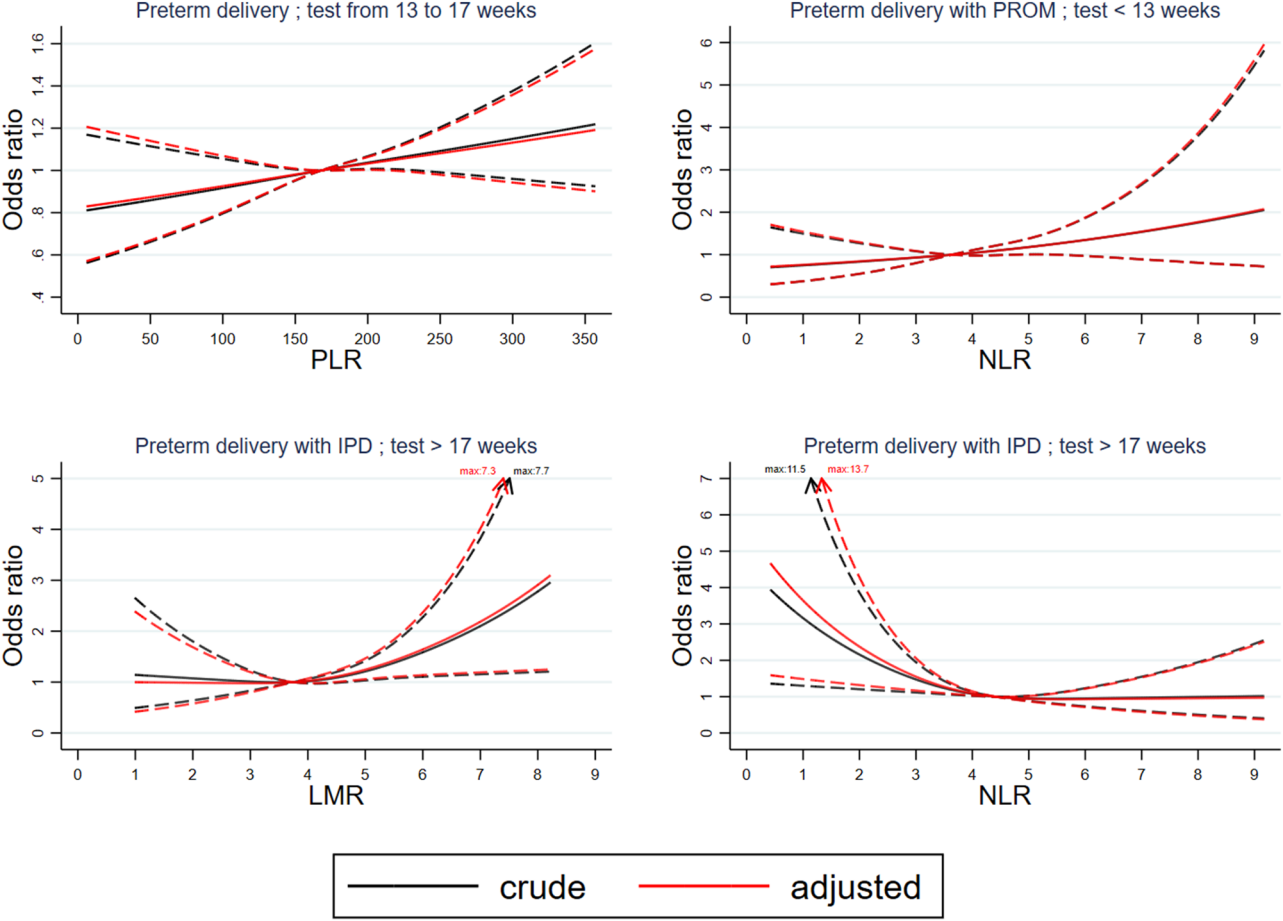
Risk of preterm delivery by blood count parameters. Reference is set at median of observed values of each parameter at each timepoint.

In Fig. 2 we show the association between LMR and NLR values measured at 18–21 weeks and risk of preterm delivery with IPD compared to when each measurement was at its median value. Women with NLR lower than 4.41 and women with LMR higher than 4.53 each had a significantly higher risk of preterm delivery with IPD compared to those who had median values of each parameter (NLR 4.41; LMR 3.75). On the other hand, although we had observed higher PLR at 13–17 weeks to be associated with higher risk of preterm delivery, we failed to detect any significant difference in risk of preterm delivery between women with extremely lower or higher values of PLR at 13–17 weeks, compared to women with median values.

Predictive values of all biomarkers at all gestational categories were low with the area under the curve (AUC) ranging from 0.50 to 0.52 (data not shown).

## Discussion

In this study including over 90,000 women, we observed the secular trends of NLR, PLR, and LMR—three biomarkers reported to reflect subclinical chronic inflammatory burden—over the course of pregnancy, as well as their values for preterm delivery.

To the best of our knowledge, our study may be the first to demonstrate the natural course these parameters take during pregnancy in a large sample of low-risk women. We found that NLR and PLR increase steadily as the pregnancy proceeds. On the other hand, LMR values were fairly stable until week 14, from when they decreased monotonically. Our findings showing that parameter values change over the course of pregnancy do not conflict with the many reports that women’s immunological and cardiovascular systems undergo a large burden when they become pregnant^17–19^. The changes in parameter values observed as pregnancy proceeds (higher NLR and PLR and lower LMR) have been associated with higher inflammation and have been reported to reflect myocardial damage and microcirculatory resistance in patients with cardiovascular diseases^10^. This may be consistent with the fact that major pregnancy complications, including IPD, tend to occur later in pregnancy as well.

We found that NLR, PLR, and LMR values differ by maternal characteristics; however, what these parameters reflect requires further investigation. Several characteristics that were associated with higher inflammation (higher NLR and PLR and lower LMR) were known risk factors for IPD, such as primiparity and higher age. On the other hand, although smoking and obesity are well known to be associated with higher inflammation, in our study such women had lower NLR and PLR values and higher LMR values, contradicting previous studies on non-pregnant individuals^20–23^. Our findings may be due to pregnant women having different mechanisms for inflammation compared to non-pregnant women, as suggested by the confusing but consistent observation that smokers have lower risk of hypertension in pregnancy^24^ although it raises blood pressure among non-pregnant individuals. Or, it may be merely reflecting the limitation of these biomarkers as markers of inflammation, as suggested from the low predictive values in our study.

In our study, high LMR and low NLR observed at 18–21 weeks were significantly associated with higher risk of preterm delivery with IPD, in line with studies suggesting that chronic low level inflammation in early pregnancy can increase risk of preterm delivery. We also observed that when women have extreme values of these parameters (LMR > 4.53 or NLR < 4.41) they have a significantly elevated risk of preterm delivery with IPD compared to women with median values, independent of known maternal characteristics. These results may help guide clinicians to identify which women need careful monitoring as they may have a higher risk in developing future complications; whole blood counts are often routinely measured during pregnancy and their ratios are easy to calculate.

However, it is important to notice that overall the predictive value was low in our study. A previous study which conducted measurements closer to delivery have observed better prognostic value of these parameters; Toprak et al. reported that PLR values at admission had an AUC of 0.62 (*p* < 0.001) for differentiating women with preterm PROM from those with spontaneous preterm labor^25^. We also observed that biomarker values before 17 weeks did not show any significant difference on risk of preterm delivery for either PROM and IPD. It is easiest to interpret that this gap in predictive value is arising from the difficulty in predicting the risk of preterm delivery early in pregnancy, compared to when symptoms are apparent.

Compared to the studies conducted in patients with malignancies^12–14^ and cardiovascular diseases^10,11^, studies on the predictive values of blood count parameters on birth outcomes are scarce. Daglar et al. reported that among women who were admitted for threatened labor, women who delivered preterm had significantly higher LMR at admission^16^. To the best of our knowledge, our study may be the first to show the association between the three parameters measured in early pregnancy and risk for preterm delivery among low-risk women. We also had sufficient sample size to conduct analyses stratified by timing of measurement of parameter values, as well as adjust for important background characteristics.

## Conclusion

In conclusion, we show that blood count parameters change over the pregnancy period; NLR and PLR increase whereas LMR decreases with increasing pregnancy age at measurement. Values also varied by maternal background characteristics. Clinicians should be aware that there is a natural trend, and that the inter-person variation is high.

## Methods

### Study sample

We used data collected by the Japan Environment and Children’s Study (JECS), a nation-wide prospective cohort study of pregnant women, their spouses, and their children in Japan. Detailed methodology has been previously reported^26,27^. In brief, pregnant women were recruited through (1) the first antenatal visit at participating health care institutions, and (2) the local government offices issuing the Mother–Child Health Handbook, from January 2011 until March 2014 in 15 Regional Centers throughout Japan. Women who were moving long-distances at the time of childbirth, who were considered to have other unavoidable circumstances preventing them from participating in the survey, or who had difficulty in filling out the questionnaires in Japanese were excluded from the study. During pregnancy, participating women were asked to fill out two questionnaires which captured demographics, life style, and behaviors as well as medical history, namely, one (M-T1) administered at recruitment, and another (M-T2) administered at mid-pregnancy. Birth characteristics and medical information were transcribed separately from medical records.

In total, 103,099 pregnancies were registered. For this study we used the dataset of the birth characteristics “jecs-ag-20160424”, which was created in April 2016 and revised in October 2016. Among 97,454 singleton pregnancies (limited to one pregnancy per woman if that woman participated multiple times in the study), 3759 miscarriages and births (including stillbirths) with unknown gestational age or born before 28 weeks, as well as 15,896 pregnancies which were missing either questionnaire or biomarker data were excluded. We conducted our analysis on 76,853 (79%) subjects, including 1678 who did not have maternal blood count results for before 22 weeks and were only included to observe the distribution of parameters by gestation (Fig. S1).

### Details of ethics approval

The Review Board on Epidemiological Studies of the Ministry of the Environment as well as the ethics committees of all the participating institutions approved the JECS protocol. We obtained written informed consent from all participants.

### Laboratory data

The pregnant participants were regularly examined in the antenatal clinic, and one blood sample per participant was drawn for maternal blood counts in routine gynecological check-ups. A vacuum sampling tube with EDTA-2Na as an anticoagulant was used for the blood sampling. The blood samples were transferred within 48 h after collection to a central laboratory where whole blood count results were obtained using automated flow cytometry, followed up by a blood smear if there were abnormal results^26^. NLR, PLR, and LMR are parameters of inflammation, with higher NLR and PLR and lower LMR indicating higher levels of inflammation. We calculated LMR, NLR, and PLR from complete blood counts using the following formulas: LMR value was calculated by dividing the absolute lymphocyte count by the absolute monocyte count, NLR value was calculated by dividing the absolute neutrophil count by the absolute lymphocyte count, and PLR value was calculated by dividing the absolute platelet count by the absolute lymphocyte count.

To construct the reference curve for the parameters we used data on all measurements (conducted for all gestations); however, for constructing predictive models, we excluded 1678 who did not have complete maternal blood count results before 22 weeks, and categorized the data by timing of measurement into the following three strata with fairly even intervals: 8–12 weeks, 13–17 weeks, and 18–21 weeks.

### Definition of variables

The primary outcome of interest was preterm delivery, defined as birth under 37 weeks of gestation. Gestational age was calculated from early ultrasound scan CRL measurement at 8–10 weeks or BPD measurements at 11–13 weeks, last menstrual period for those where early ultrasound scans were not available, or conception date for pregnancies conceived after in vitro fertilization. Due to the lack of consensus on categorization of preterm births^28^, the agreement that PROM is an distinguished cause of preterm birth, and the recent discussion that preeclampsia, small for gestational age, or placental abruption may be classified as one syndrome, we further focused on two subcategories of preterm birth: preterm delivery with premature rupture of membranes (PROM), and preterm delivery with ischemic placental diseases (IPD). We did not use the classification of spontaneous and medically induced preterm birth both because of the lack of consensus that this categorization reflects true phenotypes, as well as the fact our data, based on data transcribed from medical records at birth, lacked sufficient information to determine onset of delivery (such as whether stimulation of uterine contractions was conducted before or after onset of delivery, and whether cesarean delivery was planned or not). We defined IPD as any of the following: hypertensive disorders in pregnancy (defined as hypertension (blood pressure ≥ 140/90 mmHg) with or without proteinuria (≥ 300 mg/24 h) emerging after 20 weeks gestation but resolving up to 12 weeks postpartum), small for gestational age (defined as under 10 percent on the Japanese growth reference curve^29^, or placental abruption.

We categorized maternal socio-demographic data from the responses to questions included in the questionnaire filled out by the mother as follows: annual household income (< 2 million yen, 2–4 million yen, 4–6 million yen, 6–8 million yen, > 8 million yen, no answer), maternal education (university graduate or higher, 2-year college, vocational school, high school or less), maternal smoking status (never smoked, previously smoked but stopped before pregnancy, previously smoked but stopped because of pregnancy, current smoker), marital status (married, unmarried), and conception method (natural conception, hormone injection, in vitro fertilization). Pre-pregnancy body mass index (BMI) was calculated from height and pre-pregnancy weight (either self-reported or measured) and categorized as under 18.5 kg/m^2^, 18.5 to < 25 kg/m^2^, or ≥ 25 kg/m^2^.

Data on maternal age, parity, previous deliveries, gestational age, and infant sex were transcribed from medical records. We categorized maternal age as < 25, 25–34, or ≥ 35 and history of previous pregnancy as primipara, multipara with previous preterm birth, or multipara with no previous preterm birth.

### Statistics

First, we described the association between each of the three biomarkers (NLR, PLR, LMR) and maternal and infant characteristics. Medians and interquartile ranges (IQRs) were provided for each category, and a Wilcoxon rank-sum test was used to investigate equivalence of the median biomarker distribution across the categories.

After checking that maternal characteristics did not differ by timing of measurement, we observed the distribution of each parameter by timing of measurement. For this analysis we included measurements from all gestations (76,853 women) and calculated the IQR for each week from week 1–36 as well as constructing smoothed cubic spline models.

To investigate the predictive value of each biomarker for risk of preterm delivery, we constructed the prediction models stratified by timing of measurement (8–12 weeks, 13–17 weeks, and 18–21 weeks) as the biomarkers follow a natural trend over time. First, for each stratum, logistic regression models to estimate the association between the biomarker values and risk of preterm delivery, as well as risk of preterm delivery with PROM, and risk of preterm delivery with IPD, were constructed both before and after adjusting for background factors. For these analyses, biomarker values were log-transformed prior to analysis.

Next, for biomarkers where we observed a significant association in the logistic models we constructed spline curves to illustrate how risk changed with biomarker value, with the reference set at the median of the observed values and adjusting for other background factors.

Stata software (StataCorp. 2015. Stata Statistical Software: Release 14. College Station, TX: StataCorp LP.) was used for all analyses.

## Supplementary Information

Supplementary Information.
