# Supplementary material for: Maternal blood count parameters of chronic inflammation by gestational age and their associations with risk of preterm delivery in the Japan Environment and Children’s Study
Source: Sci Rep. 2021 Jul 30;11:15522. doi: 10.1038/s41598-021-93101-2 (PMC8324902; doi:10.1038/s41598-021-93101-2)
Supplement: Supplementary file 1 — Supplementary Information. [file 41598_2021_93101_MOESM1_ESM.docx]

Figure S1 Population Flow Chart

| 103,099 | Pregnancies | |  |
| --- | --- | --- | --- |
|  | 5645 | Data of women with second and third agreement to participate | |
|  | 1907 | Multiple births and births missing record on multiplicity | |
| 97,454 Singleton pregnancies | | | |
|  | Extremely preterm pregnancies and those with unknown gestational age | | |
|  | 1451 | <28 weeks | |
|  | 2308 | Missing gestational age | |
|  | Missing questionnaires | | |
|  | 2118 | Non-response to questionnaire (M-T1, M-T2) | |
|  | Blood test | | |
|  | 10,041 | No blood test done | |
|  | 3737 | Missing data for one of biomarkers | |
|  | 1678 | Biomarkers recorded after 22 weeks or before 6 weeks | |
| 75,175 women included in sample | | | |
